# Supplementary figures and images for: The combined action of CTCF and its testis-specific paralog BORIS is essential for spermatogenesis
Source: Nat Commun. 2021 Jun 22;12:3846. doi: 10.1038/s41467-021-24140-6 (PMC8219828; doi:10.1038/s41467-021-24140-6)

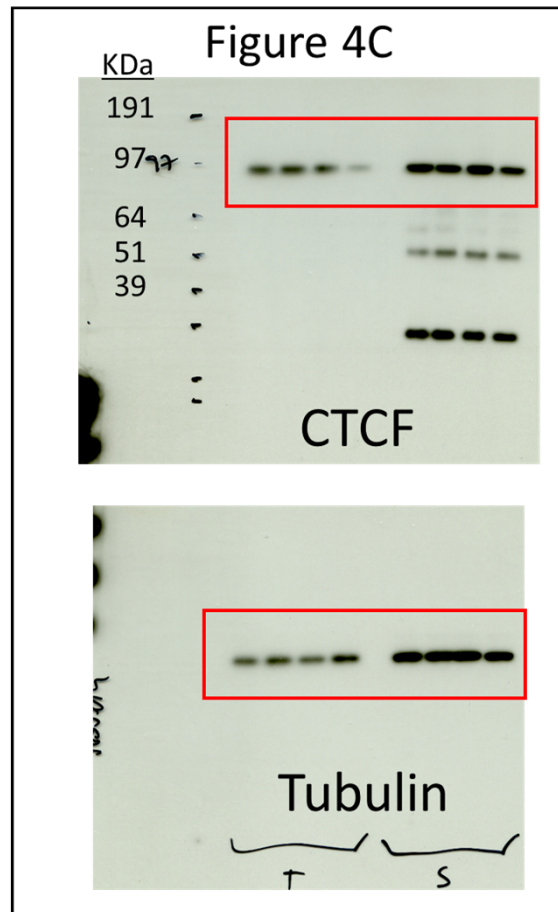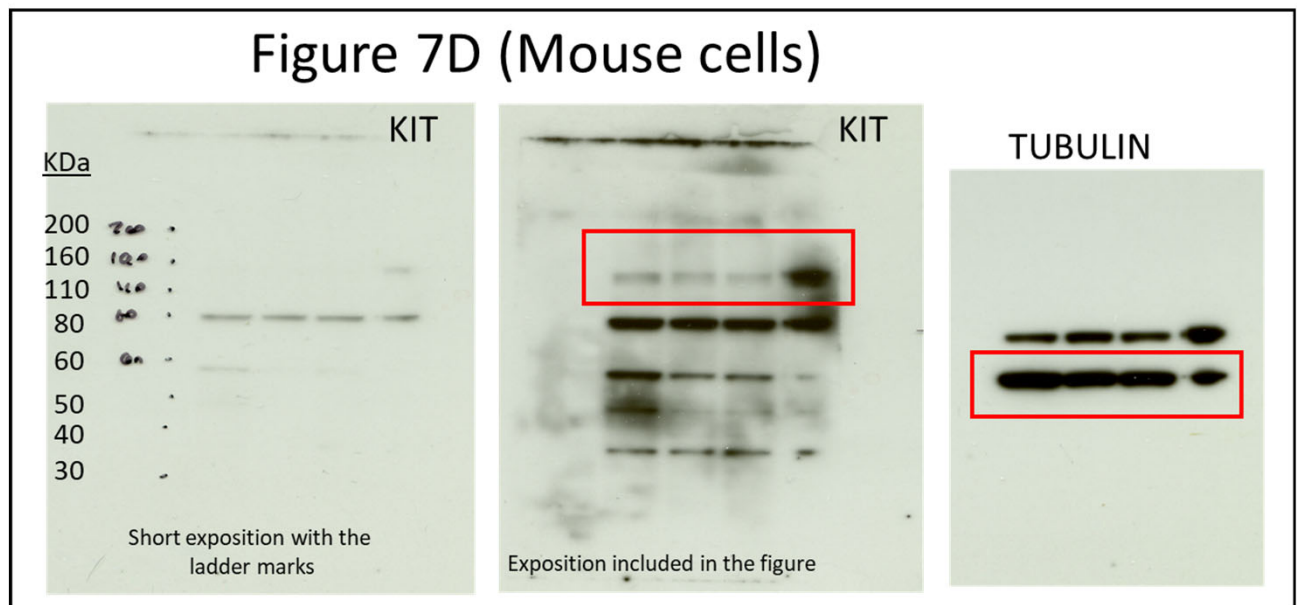

Figure 7E

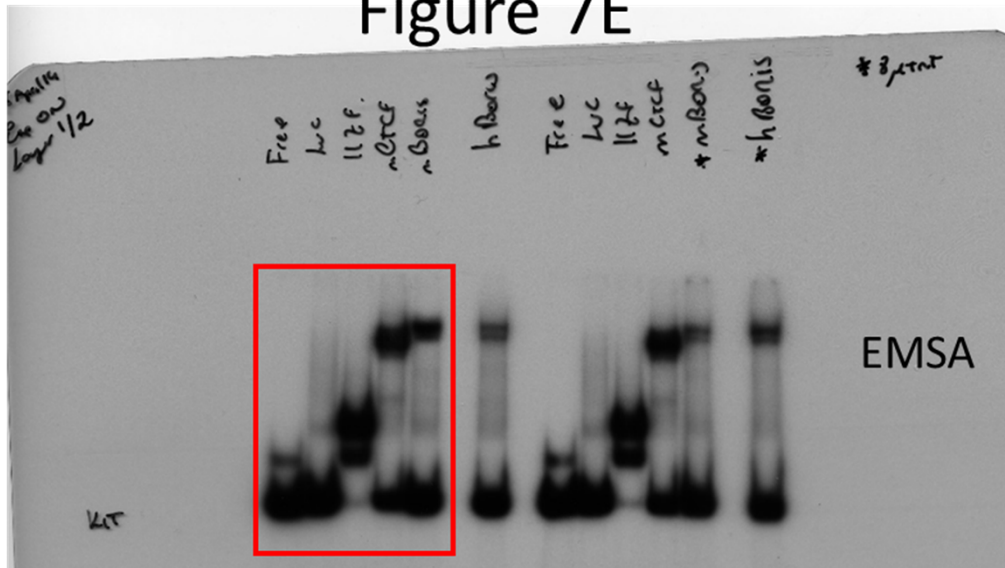

Figure 7H (Human cells)

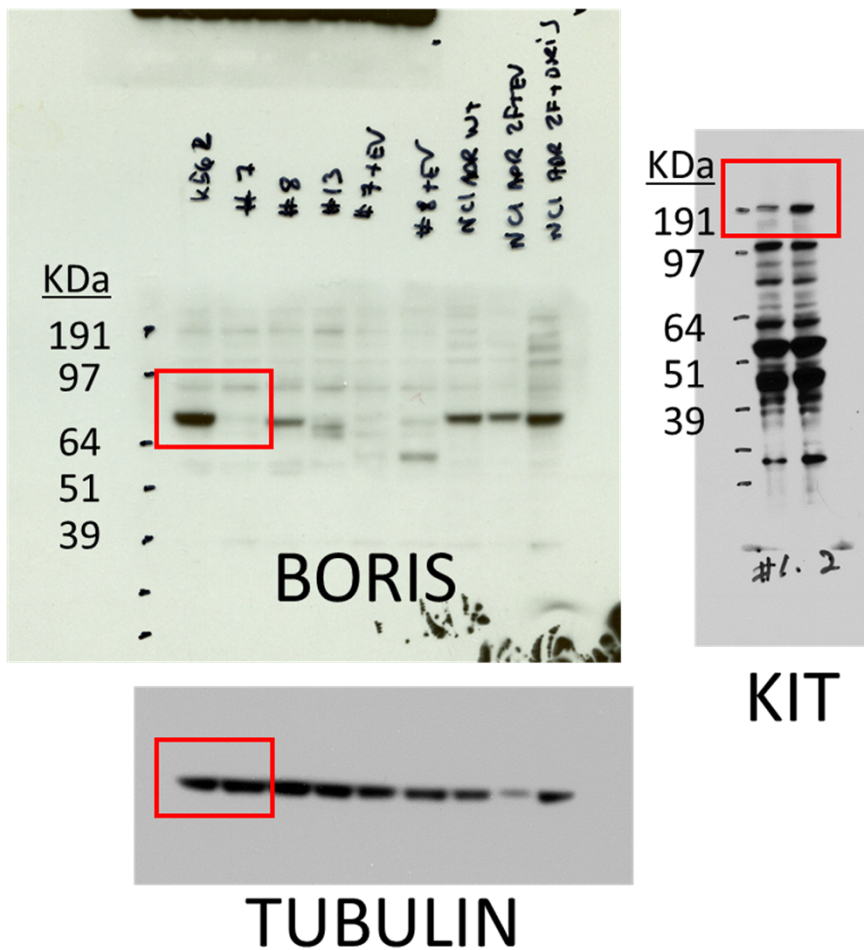

Supplement: Supplementary file 9 — Source Data [file 41467_2021_24140_MOESM9_ESM.zip › Source_data_and_uncropped_images/Uncropped film images.pdf]
